# Temporal Sequence of Visuo-Auditory Interaction in Multiple Areas of the Guinea Pig Visual Cortex

**DOI:** 10.1371/journal.pone.0046339

**Published:** 2012-09-28

**Authors:** Masataka Nishimura, Wen-Jie Song

**Affiliations:** Department of Sensory and Cognitive Physiology, Graduate School of Medical Sciences, Kumamoto University, Kumamoto, Japan; University of Chicago, United States of America

## Abstract

Recent studies in humans and monkeys have reported that acoustic stimulation influences visual responses in the primary visual cortex (V1). Such influences can be generated in V1, either by direct auditory projections or by feedback projections from extrastriate cortices. To test these hypotheses, cortical activities were recorded using optical imaging at a high spatiotemporal resolution from multiple areas of the guinea pig visual cortex, to visual and/or acoustic stimulations. Visuo-auditory interactions were evaluated according to differences between responses evoked by combined auditory and visual stimulation, and the sum of responses evoked by separate visual and auditory stimulations. Simultaneous presentation of visual and acoustic stimulations resulted in significant interactions in V1, which occurred earlier than in other visual areas. When acoustic stimulation preceded visual stimulation, significant visuo-auditory interactions were detected only in V1. These results suggest that V1 is a cortical origin of visuo-auditory interaction.

## Introduction

According to the classical view of sensory cortices, sensory information from each modality is initially sent to a specific primary sensory area, while the convergence of different modalities occurs only in nonprimary areas [1]. Recent studies in humans, monkeys, and ferrets have reported that sensory responses at the early stage of hierarchical cortical processing, including those in the primary sensory cortices, are influenced by extramodal stimulation [2], [3], [4], [5], [6], [7], [8], [9], [10], [11], [12] (for reviews see Ref. [13], [14], [15], [16], [17]). In monkeys, for example, auditory stimulation that is simultaneously presented with visual stimulation modulates visual responses in the primary visual cortex (V1) [10]. In humans, visuo-auditory interactions in V1 have been detected in magnetoencephalography (MEG) and functional magnetic resonance imaging (fMRI) studies [3], [12]. Raij et al. also reported acoustically evoked responses in the human calcarine fissure [12]. Anatomically, projections from auditory cortices to V1 have been demonstrated [18], [19], [20], [21], although these projections appear sparse.

According to anatomical evidence, visuo-auditory interactions in V1 could originate within V1. Alternatively, sparse projections from auditory cortices to V1 might not impart a significant functional impact, and the visuo-auditory interactions reported in V1 are actually due to feedback from interactions in the extrastriate cortex. To discriminate between these possibilities, the present study analyzed the temporal order of visuo-auditory interactions in cortical visual areas. Accordingly, an optical imaging technique using voltage-sensitive dyes was used to record visual and auditory responses. This imaging technique permits recording of neural activity from a large cortical area, including multiple visual areas and an auditory area, at a high spatiotemporal resolution (spatial resolution: 62.5 µm × 62.5 µm; temporal resolution: up to 1 ms) [22], [23], [24], [25], [26], [27], [28]. Our results supported the hypothesis that V1is the initial site for visuo-auditory interactions in the cortex.

## Results

Results were obtained from 35 albino Hartley guinea pigs and four pigmented ones. All optical imaging was performed on the left hemisphere.

### Identification of Subfields in the Visual Cortex

**Figure 1:**
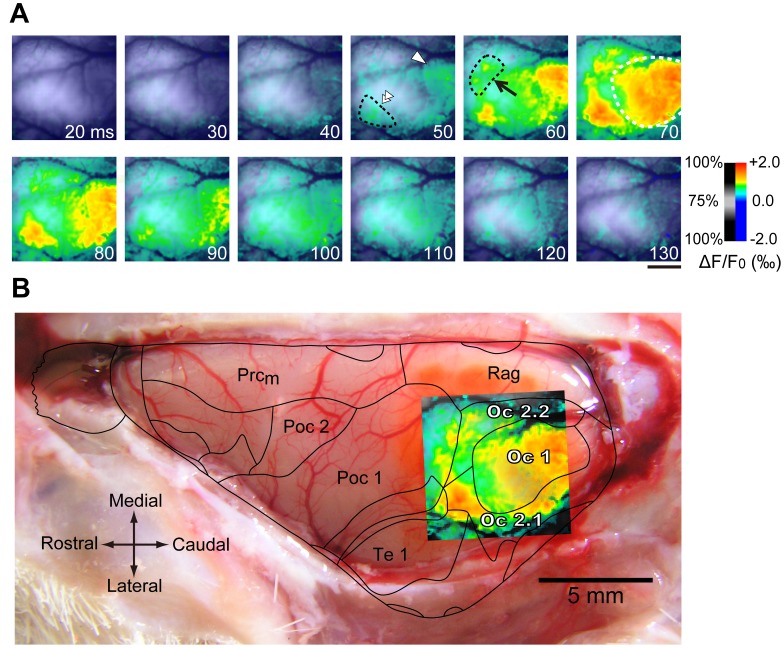
Identification of visual areas in the guinea pig occipital cortex. (A) A time-series of frames showing color-encoded cortical activity responding to a flash light stimulus in the left occipital cortex. Color-encoding used the color bar shown to the right, and the encoded color image was superimposed on the cortical surface with transparency defined by the transparency bar shown next to the color bar. The white number in each figure represents time after stimulus onset in ms. At 50 ms after stimulation, responses were observed in a caudal area (white arrowhead in the fourth graph of the upper row) and in a rostrolateral area (double white arrowhead). At 60 ms after stimulation, responses became evident in a rostromedial area (arrow in the fifth graph of the upper panel). The response peaked around 70 ms and declined thereafter. Scale bar is 2 mm. (B) Superimposition of cortical activity on the surface of the left hemisphere, which was exposed after recording. To identify visual areas, borders between cortical areas reported in guinea pigs by Wree et al. [29] were also superimposed. It is clear from the figure that the majority of the caudal responding area corresponds to occipital area 1 (Oc1), the rostrolateral responding area largely corresponds to a rostral portion of Oc2.1, and the rostromedial responding area largely corresponds to a rostromedial portion of Oc2.2. In the subsequent figures, we refer to the caudal area as V1, the rostrolateral area as LV, and the rostromedial area as RV. Other abbreviations follow Wree et al. [29]. Scale bar is 5 mm.

Responses to the visual stimulus (50 Lux diffused light flash, 10 ms duration) were optically recorded from the visual cortex, which was stained with RH-795 (Fig. 1). Spatiotemporal changes in optical signals were coded in pseudo-color, as illustrated in Figure 1A. Approximately 50 ms after stimulus onset, two spatially isolated areas (caudal and lateral, which responded to stimuli), were clearly observed (white arrowhead and white double arrowhead in Fig. 1A). After 10 ms, another spatially isolated area (rostromedial area) was also observed (arrow in Fig. 1A). At approximately 70 ms after stimulus onset, the caudal responding region expanded, with a moderate preference towards the rostrocaudal direction. After the appearance of maximal expansion of responding areas, the responses gradually became smaller over time in all areas (Fig. 1A, lower panel). To identify V1, the spatiotemporal pattern of visual responses at the time of maximal expansion was superimposed on the cortical surface using blood vessels as landmarks (Fig. 1B). Additionally, the histological parcellation of cortical areas in guinea pigs from a previous publication [29] was superimposed on the cortical surface (Fig. 1B). As seen in Fig. 1B, most of the caudal area that responded to the visual stimulus was within the occipital area 1 (Oc1) or V1, except the lateral edge. In addition, the lateral area was within the rostromedial part of occipital region 2.1 (Oc2.1), and the rostromedial area comprised a large rostral part of occipital region 2.2 (Oc2.2). We refer hereafter to the lateral area as the lateral visual area (LV) and to the rostromedial area as the rostral visual area (RV), respectively. The locations of LV and RV indicate their extrastriate nature.

### Visual Responses in Visual and Auditory Areas

**Figure 2:**
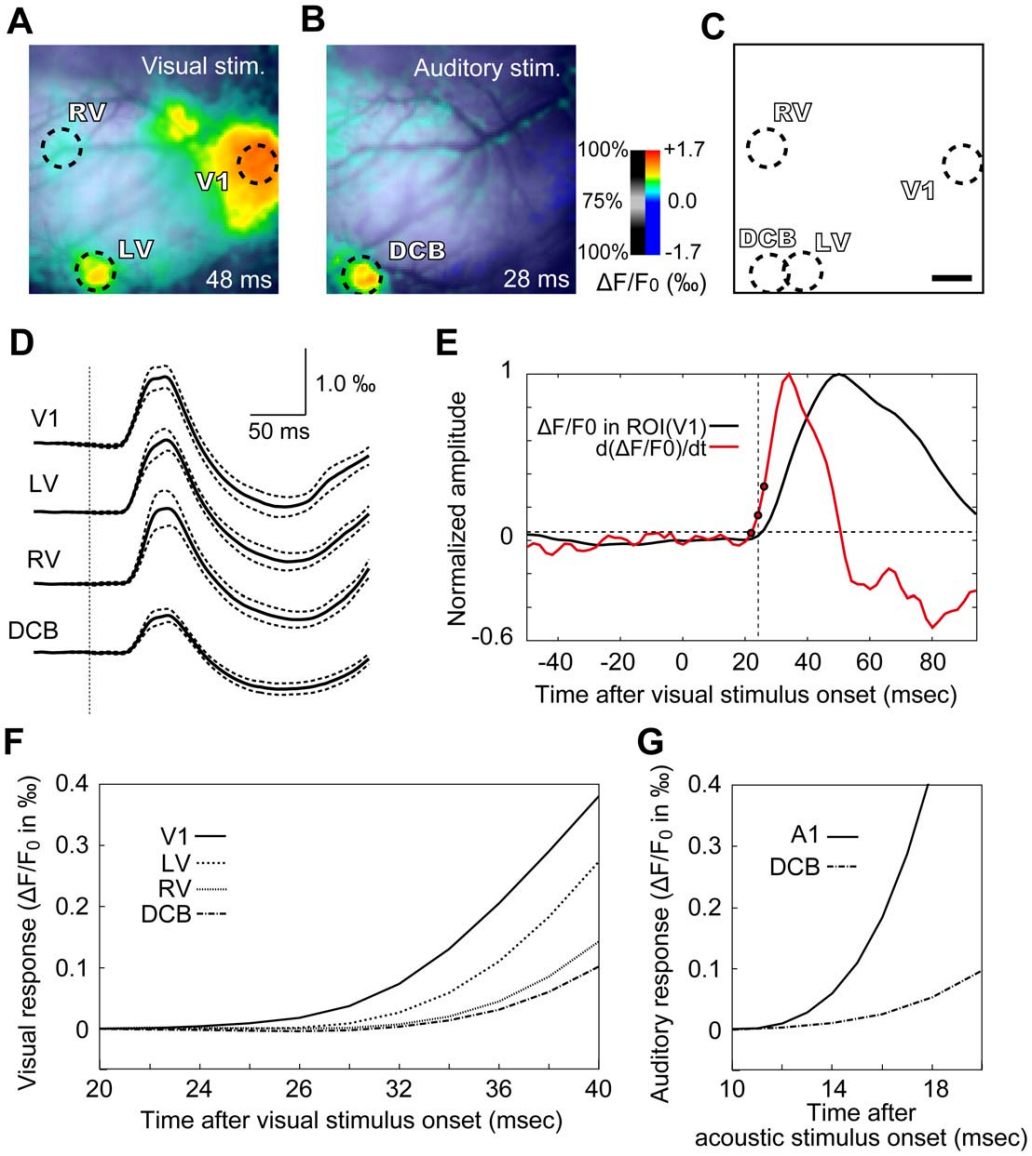
Visual responses and onset latencies in cortical fields. (A) Cortical response to visual stimulation at 48 ms after stimulus onset. Responses in the V1, LV, and RV areas were readily recognizable, and the three areas were well separated from each other. A circular ROI with a diameter of 1 mm was set in each of the subfields to include the response epicenter and to cover as many responsive pixels as possible. (B) Response to auditory stimulation at 28 ms after stimulus onset; recorded in the same cortical field as in (A). Response in the dorsocaudal belt portion of the auditory cortex (DCB) is readily recognizable. An ROI was set on the DCB similar to the visual fields. (C) Summary illustration of ROIs in the recording field. Note the partial overlap between DCB and LV. (D) Responses to visual stimulation in all pixels within an ROI were averaged for each animal, and data from 10 animals were then pooled together. The black trace is the population mean and the dotted lines represent SEM. The vertical dotted line marks the onset time of visual stimulus. (E) The averaged visual response in the ROI of V1 in one animal (the black trace) is shown together with its differentiated trace (the red trace). The dotted line marks the sample point (the middle circle) at which the differentiated signal first exceeded baseline level. (F) The mean traces in (D) are shown at a higher magnification, illustrating that visual responses occurred in the order of V1, LV, RV, and DCB. (G) For comparison, the population average of auditory responses in A1 (n = 6) and in DCB (n = 10) are shown. Scale bar in (C) is 1 mm and also applies to (A) and (B). Other conventions follow Fig. 1.

To characterize responses from each area, a region of interest (ROI) was assigned to V1, LV, and RV, respectively (Fig. 2A, visual responses at 48 ms). The recording field also contained a dorsocaudal belt portion of the auditory cortex (DCB) [30], which was identifiable by its robust response to acoustic stimulation (Fig. 2B, auditory response in the same cortical area as in Fig. 2A; acoustic stimulation: 50 ms broadband noise). The DCB partially overlapped with the LV in all animals examined (Fig. 2C). ROIs in all visual areas, as well as in the DCB, had a diameter of 1 mm, with the center of the ROI in each area set by eye to contain the response epicenter and to cover as many responding pixels as possible (Fig. 2A, B). A 0.2 mm shift in the ROI center did not qualitatively alter the results. Cortical responses were averaged among all pixels within each ROI. Fig. 2D shows the time course of responses to visual stimulation in each area. The voltage-sensitive dye RH-795 converts membrane depolarization to a fluorescence reduction, which has been conventionally depicted as a positive change in previous studies [22], [25], [31], [32], [33], [34]. The present study followed the same convention. In visual areas, the response to visual stimulation was a transient positive change followed by a slower negative change (Fig. 2D). The responses had bimodal peaks in most animals (n = 9 out of 10; Fig. 2D), in agreement with a previous study in rats [35]. Similar responses to the visual stimulus were also observed in the DCB, although the amplitude was smaller (Fig. 2D, bottom trace). To estimate visual response latencies, the spatially averaged signal in the ROI of each area was differentiated using the equation d(ΔF/F_0_)/dt[n] = ΔF/F_0_[n]–ΔF/F_0_[n–1], where n is the sample number. The response latency was measured as the time interval from stimulus onset to when the differentiated signal exceeded the maximal baseline value (baseline = 100 ms recording prior to stimulus onset; Fig. 2E; referred to as the differentiation-thresholding method). The visual response latencies in V1, LV, RV, and DCB were 24.7±1.1 ms, 29.3±0.9 ms, 30.9±1.0 ms, and 32.5±1.1 ms, respectively (n = 9, error is SEM; Fig. 2F). These estimated latencies may have been affected by response amplitude, because smaller amplitude leads to an apparent slower rise of signal and may thus result in estimation of a longer latency. To test this issue, we measured response latencies after normalization with response peak amplitude, using 1% as threshold. Before normalization, we subtracted the average of signals in 10 ms after stimulation (before response onset), to suppress slow fluctuations. Nevertheless, the baseline fluctuation before stimulation was occasionally higher than the 1% threshold. To measure the response latency and to avoid measuring the latency of a fluctuation, for each recording we searched sample by sample, starting from the response peak towards the beginning of the recording, to find the last sample that keeps a value above the threshold; the interval from the sample to the stimulus onset was then measured as response latency. The threshold of 1% of peak amplitude, rather than a threshold that exceeds baseline fluctuation, was used to avoid overestimation of response latencies. As a result, the latencies estimated for normalized responses to visual stimulation in V1, LV, RV, and DCB were 25.8±1.8 ms, 29.1±2.0 ms, 31.1±3.0 ms, and 30.5±2.6 ms, respectively. These values are close to the latencies measured by the differentiation-thresholding method.

For comparison, we also measured auditory response latencies in the primary auditory cortex (A1) and in the DCB. The latency was 12.2±0.3 ms in A1 (n = 6), and 15.2±0.4 ms in DCB (n = 10) (Fig. 2G).

### Auditory Responses in the Identified Cortical Areas

**Figure 3:**
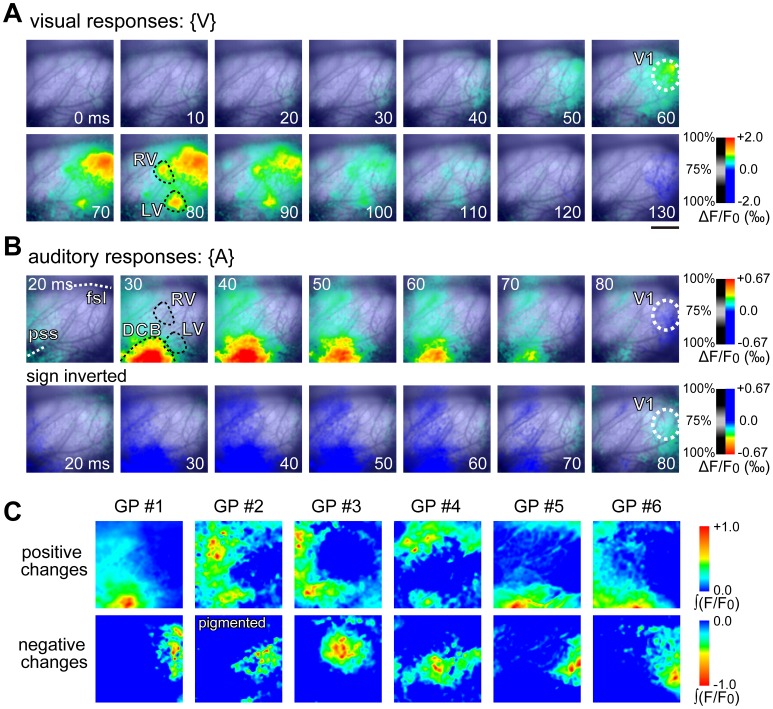
Spatiotemporal patterns of auditory responses in the visual cortex. (A) Responses to visual stimulation were recorded initially for the identification of visual areas. Three areas, the V1, the LV, and the RV, are identifiable. The rostrocaudal distance between the V1 and the other areas appears shorter in this figure; this is because LV and RV are labeled at a later time of 80 ms, at which time V1 activity had spread. (B) Upper row: spatiotemporal patterns of auditory responses in the cortex, recorded in the same field as in (A). The strongest response was observed in DCB. The visual area contours found in (A) are superimposed. Weak positive responses are detected in LV and RV. However, within V1, only a late negative response is observed. This is better illustrated when the sign of the signal is reversed (lower row). fsl: fissura sagittalis lateralis; pss: pseudosylvian sulcus. (C) Upper row: positive auditory responses in a time-window between 0 ms and 70 ms after stimulus onset are integrated and normalized by the maximum for each animal. The normalized values are color-coded with the color bar shown to the right. Results are from six animals, all of which are albinos except GP #2. Lower row: negative auditory responses in the same six animals in a time-window between 0 ms and 70 ms after stimulus onset are integrated, normalized, and illustrated. It is clear from the figures that V1 exhibits negative signals, while the other visual areas and DCB exhibit positive signals. The integrated positive signals and negative signals also appear to be spatially complementary. Scale bar is 2 mm in (A) and also applies (B) and (C).

**Figure 4:**
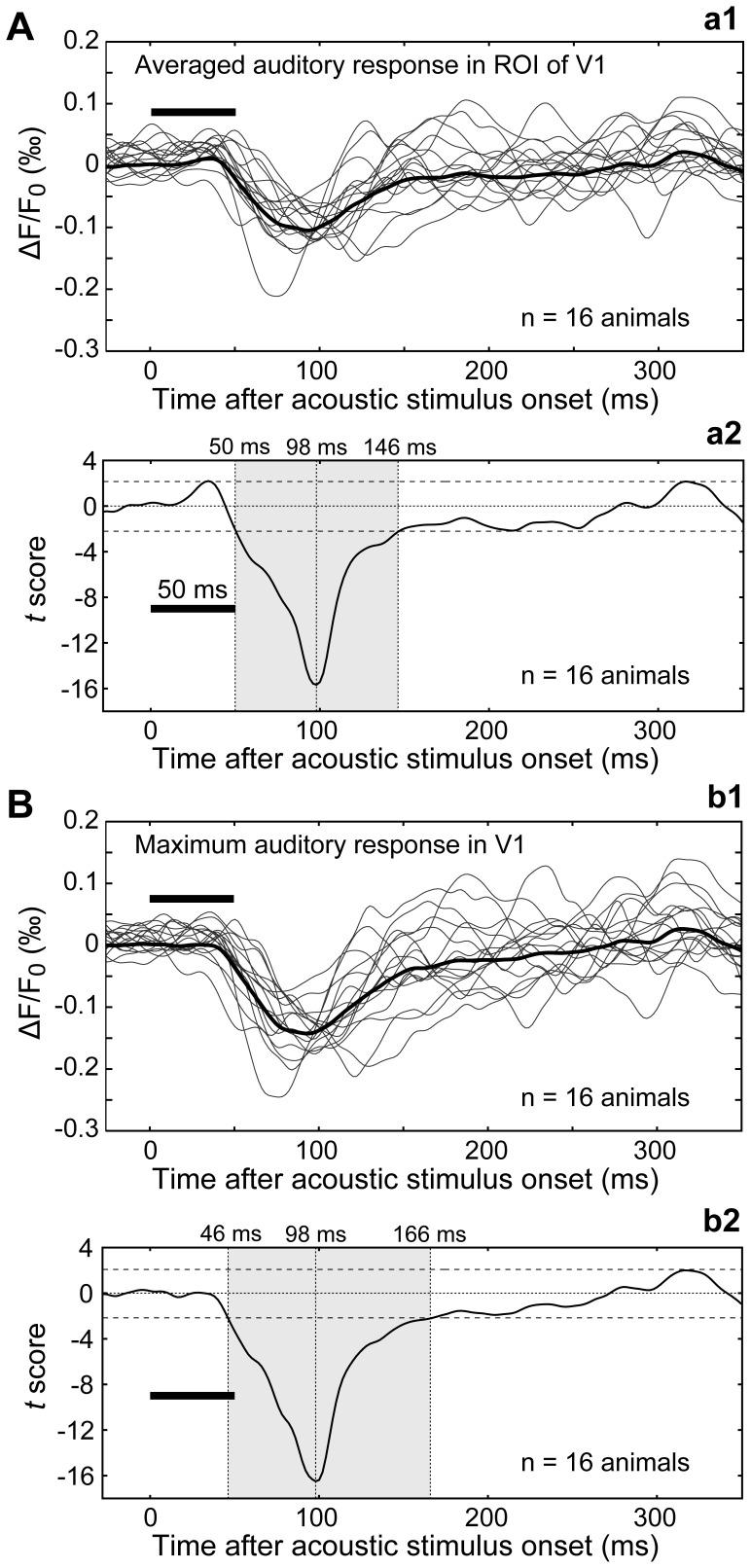
Waveforms of auditory response in V1. (A) Averaged auditory responses in the ROI of V1. (a1) Each thin trace is the response in V1 in a single animal; the trace is the average of 96 trials. The thick trace is the average of all thin traces obtained from 16 animals. (a2) From the data shown in (a1), a *t*-score was calculated at each sampling point, and all calculated *t*-scores are presented as a time series. The shaded region shows the time window when responses are significantly negative (*p*<0.05; Student's *t*-test). The horizontal bar shows timing and duration of the auditory stimulus. (B) The auditory response at the epicenter of negative signals in V1. (b1) Each thin trace is the response in V1 in a single animal; the trace is the average of 96 trials. The thick trace is the average of all thin traces obtained from the same 16 animals as in (a1). (b2) From the data shown in (b1), a *t*-score was calculated at each sampling point, and all calculated *t*-scores are presented as a time series. Other conventions follow (a2).

**Figure 5:**
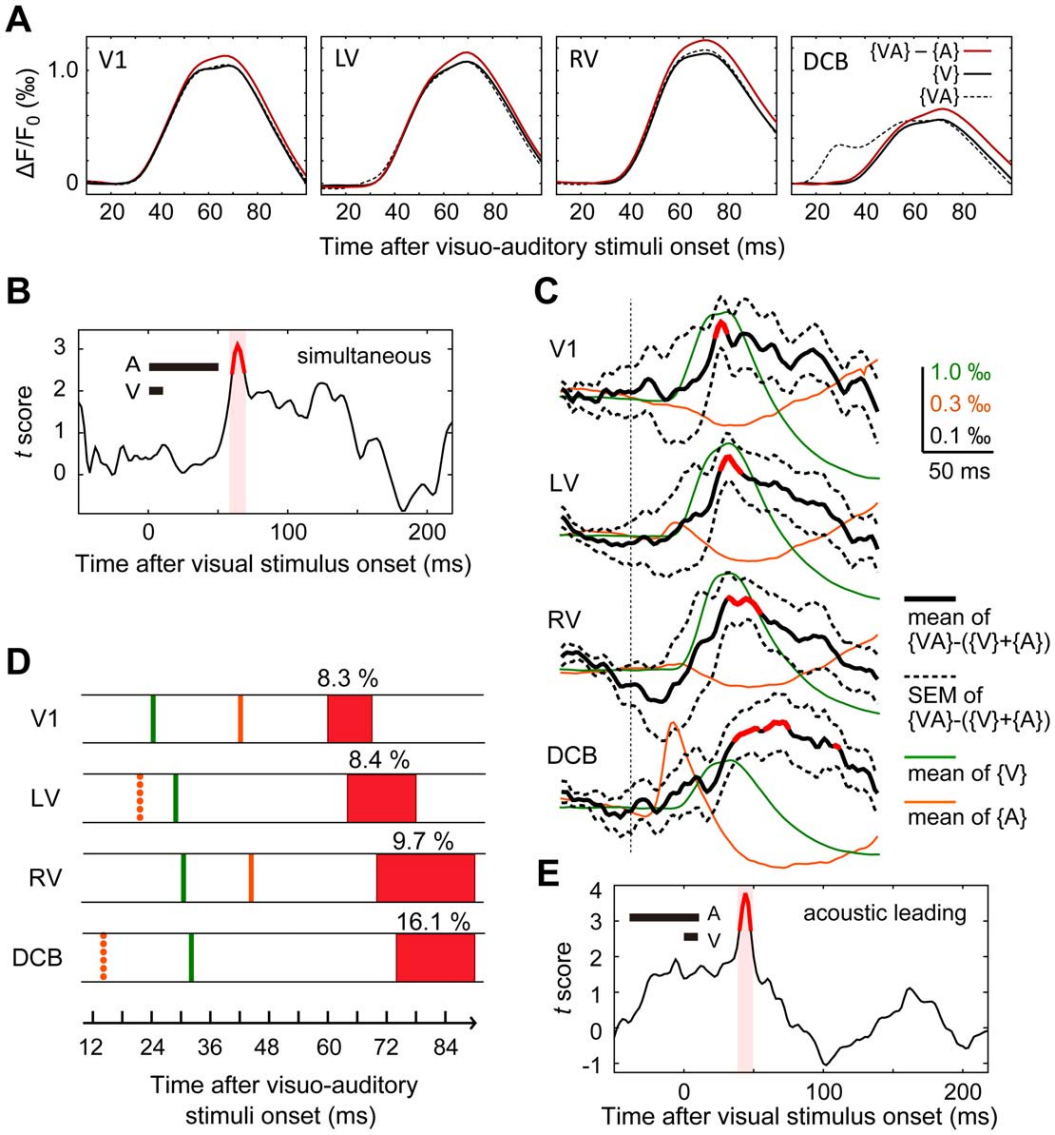
Cortical visuo-auditory interactions occurred initially in V1. (A) The averaged responses (average in the ROI of each animal followed by average among animals) to visual stimulation {V} (the black trace), auditory stimulation {A}, and combined presentation of visual and auditory stimuli {VA} (the dotted trace), were obtained from 10 animals in different cortical fields. The larger amplitude of ({VA}−{A}) (the red trace) compared to {V} suggests visuo-auditory interaction. (B) Visuo-auditory interactions estimated by {VA}−({V}+{A}). A *t*-score for {VA}–({V}+{A}) was calculated at each sampling point, and all calculated *t*-scores are presented as a time series. Shown here are V1 results, with simultaneous presentation of visual and auditory stimuli (simultaneous condition). The reddish shaded region shows the time window during which {VA}–({V}+{A}) is significantly different from zero (*p*<0.05; Student's *t*-test). (C) Comparison of visuo-auditory interactions in different areas in the simultaneous condition. The green trace in each figure is the average of {V} (average in the ROI of each animal followed by average among animals), and the orange trace is the average of {A}. The average of {VA}−({V}+{A}) is shown as a black trace, and the SEM of {VA}−({V}+{A}) is illustrated as a dotted line. The black trace is drawn in red if {VA}–({V}+{A}) is significant, as tested in the way shown in (B). It is clear from the figure that significant visuo-auditory interaction occurs in all visual and auditory areas, and the interaction occurs initially in V1. Calibration scales apply to all traces. (D) Schematic illustration of visual response latencies (green lines) and auditory response latencies (orange lines; solid line represents negative response, and dotted line represents positive response), as well as the time window of significant visuo-auditory interaction (red) in the simultaneous condition. The number above each red box is the average of the ratio of ({VA}–{A}) to {V} in the time window. (E) Time series of *t*-score for {VA}–({V}+{A}) in V1, when auditory stimulation preceded visual stimulation. In this stimulation condition, significant {VA}–({V}+{A}) was observed only in V1.

Following identification of V1, LV, and RV through the use of the visual stimulus (Fig. 3A), broadband noise (50 ms in duration) was binaurally applied to test if auditory responses could be evoked in the visual areas. To acoustic stimuli, both positive changes and negative changes in the optical signal were observed from different areas in the recording field (Fig. 3B). As expected, the largest positive change was observed in the DCB (Fig. 3B), and a positive change was also consistently observed in LV (Fig. 3B). Positive signals in RV, however, were weak (Fig. 3B). The average peak positive response from 10 animals was 0.031%, 0.005%, and 0.002% in the DCB, LV, and RV, respectively. The LV response was 16% of the DCB response, and the RV response was only 6% of the DCB response. In addition, positive signal changes were observed in the area rostral to RV in all animals, as well as in the area medial to the fissura sagittalis (fsl) in some animals (Fig. 3B). Similar to visual responses, the positive auditory response in LV, RV, and DCB was followed by a slower negative change (see below). In V1, a significant auditory response was also found in 23 of 27 animals examined (*p*<0.05; Student’s *t*-test for difference from baseline level). In all animals that exhibited a significant auditory response, only a negative change was observed without detectable preceding positive changes (Fig. 3B, Fig. 4, Fig. 5C). The average peak negative response from 10 animals was 0.010% in the V1, 0.009% in LV, 0.007% in the RV, and 0.020% in the DCB. Because of the influence of the preceding positive response, the negative peaks should not be taken at face value; nevertheless it was clear that like the positive response, the negative response from visual areas was much weaker than in the DCB.

To further illustrate the spatial distribution of positive and negative signals in the recorded area, all positive and negative signals were integrated for each pixel, respectively, over the time window from stimulus onset to 70 ms after onset, and the values were normalized with the maximum (maximum among pixels; Fig. 3C). As can be seen in Figure 3C, positive signals were located at peripheral regions of the recorded area except the caudal portion, while the majority of negative signals were observed within V1. The negative V1 signal was spatially complementary to positive changes in other areas, which was a consistent observation among the animals (Fig. 3C). No obvious difference was detected between pigmented (GP #2 in Fig. 3C) and albino animals.

To examine the temporal features of V1 auditory responses, we first applied the differentiation-thresholding method to measure the latency of averaged responses in the ROI in V1. An average latency of 44.8 ms ±0.8 ms (SEM) was detected in 16 of the 23 animals that showed an auditory response (Fig. 4Aa1). The latency measured after normalization, using -1% as threshold, had similar values (42.8±2.0 ms, n = 16; *p*>0.05; Student’s *t*-test). Using data from the 16 animals, we also tested when the auditory responses reached significance after stimulus onset. Accordingly, we calculated a sequential *t*-score to determine the time window of significant auditory response (Fig. 4Aa2). The response reached significance at 50 ms after stimulus onset, peaked at 98 ms, and lasted for 96 ms (Fig. 4Aa2; *p*<0.05; Student’s *t*-test). We further examined auditory response latency in V1 using the maximal response (maximum among pixels) in each of the 23 animals. An average latency of 42.8±1.0 ms was detected using the differentiation-thresholding method, in the same 16 animals as in the case of the ROI average analysis (Fig. 4Bb1). Again, the latency measured after normalization had similar values (39.4±3.4 ms, n = 16; *p*>0.05; Student’s *t*-test). Statistical tests revealed that the maximal response reached significance at 46 ms, peaked at 98 ms, and lasted for about 120 ms (Fig. 4Bb2; *p*<0.05; Student’s *t*-test).

### Visuo-auditory Interactions in Visual and Auditory Areas

The above results demonstrated that all examined cortical visual areas, including V1, responded to the acoustic stimulus. To test the influence of the acoustic stimulus on visual responses in the visual areas, responses to visual, auditory, and combined stimuli were recorded and denoted by {V}, {A}, and {VA}, respectively. The presentation order of the stimuli was interleaved to minimize the effect of response temporal fluctuations over the recording period. Again, responses in the ROI of each field were spatially averaged. To test dependence of visuo-auditory interactions on the relative start timing of auditory and visual stimuli, the acoustic stimulus was initiated either simultaneous to the visual stimulus (referred to as “simultaneous condition”), or 40 ms prior to the onset of the visual stimulus (referred to as “acoustic leading condition”).

In the simultaneous condition, as shown in Fig. 5A, the magnitude of ({VA}−{A}) was larger than {V} at times around the peak of {V}, in all fields examined, suggesting an enhancement of visual response by acoustic stimulation. In previous investigations [2], [36], the visuo-auditory interaction was evaluated by calculating the difference between the response evoked by the combined visual and auditory stimuli, {VA}, and the sum of the visual response and auditory response ({V + {A}), *i.e*., {VA}− ({V}+{A}). Following this method, we calculated the differences in a population of animals. The results were then used to calculate a sequential *t*-score to determine the time window of significant visuo-auditory interaction (Fig. 5B). Significant visuo-auditory interactions were found in all identified cortical areas (*p*<0.05; paired *t*-test; n = 10; Fig. 5C). In V1, the interaction reached significance at 60 ms and peaked at 64 ms (*p*<0.05; paired *t*-test). In LV, the interaction reached significance at 64 ms and peaked at 72 ms (*p*<0.05; paired *t*-test). In RV, the interaction reached significance at 70 ms and peaked at 84 ms (*p*<0.05; paired *t*-test), and in the DCB the interaction reached significance at 74 ms and peaked at 84 ms (*p*<0.05; paired *t*-test).

Estimation of the time at which visuo-auditory interaction reached significance depends on the signal-to-noise ratio (SNR) of the recordings. A lower SNR will lead the interaction to reach significance at later time points. To test whether the estimations made above were affected by differences in the SNR, we measured SNR at the peak of {VA}− ({V}+{A}) in each cortical field. SNR was defined as peak amplitude divided by the standard deviation at peak time. The SNRs in V1, LV, RV, and DCB were 0.90, 0.90, 0.91, and 0.91, respectively. The small difference among these values suggests that the SNR had little effect on the difference in the estimated time when visuo-auditory interaction reached significance, in different cortical fields.

Figure 5D shows onset time of visual and auditory responses, as well as the time window of significant visuo-auditory interactions. Significant visuo-auditory interaction always occurred at times close to the positive peak of the visual response, or the negative peak of the auditory response, not at the onset of responses (Fig. 5C, Fig. 5D). During the time interval of significant visuo-auditory interaction, the average enhancement of ({VA}–{A}) compared to {V} was close to 10% for visual areas and 16.1% for the DCB, respectively (Fig. 5D).

In the acoustic leading condition, a significant visuo-auditory interaction was observed only in V1, and the interaction reached significance at 40 ms after visual stimulus onset (*p*<0.05; paired *t*-test) and peaked at 44 ms (*p*<0.01) (Fig. 5E, n = 10). Therefore, when the acoustic stimulus was presented 40 ms prior to the visual stimulus, visuo-auditory interaction occurred 20 ms earlier than the simultaneous condition.

## Discussion

The present study is the first to analyze auditory responses and visuo-auditory interactions in both the striate and extrastriate cortex, through the use of optical imaging with a voltage-sensitive dye. Our results demonstrate that the striate and extrastriate regions in guinea pigs exhibit responses to auditory stimulation. When visual and auditory stimuli were presented simultaneously, significant visuo-auditory interactions were observed in all visual areas, as well as in an auditory area. The interaction in V1 occurred earlier than in all other areas. In the acoustic leading condition, significant visuo-auditory interactions were observed only in V1. These results suggest that V1 is a cortical origin of visuo-auditory interaction.

### Auditory Response in Visual Areas

Three isolated activated areas were detected in the occipital cortex of guinea pigs following a flash of diffuse light, which led to the identification of three visual fields (V1, LV, and RV). The identification was based on comparisons with previous histological examinations of guinea pig cortical areas [29]. The localization of V1 in Oc1 suggests its nature of striate cortex, while the localization of LV and RV in Oc2 suggests their nature of extrastriate cortex. The notion that LV and RV are extrastriate cortices is also supported by a previous neuronal tracing study [37]. Spatz et al. [37] have shown that extrastriate fields in guinea pigs stay in two clusters: one in the area rostrolateral to V1 and the other rostromedial to V1. RV thus corresponds to the rostromedial cluster and LV corresponds to the rostrolateral cluster.

The V1 identified here exhibited shorter response latency compared to LV and RV, which is consistent with previous reports in macaques showing that V1 responded earlier to a visual stimulus than extrastriate areas [38], [39]. These observations further support our identification of LV and RV as extrastriate areas. The earlier onset of response in V1 compared to the secondary visual area (V2) has also been reported in a voltage-sensitive-dye imaging study in rats [40]. Xu et al. [40] reported an interesting observation that a visually-evoked activity-wave in V1 is compressed at the V1–V2 border before propagating into V2, resulting in a delay (35 ms) of activity in V2. In the present study, we found no wave compression and the response onset latency in extrastriate cortex was longer than that in V1 by only a few ms. The reason for this discrepancy between studies is not clear, but it might be attributable to differences in anesthesia used (isoflurane vs. a mixture of ketamine and xylazine), visual stimulus (drifting grating vs. flash light), and animal species (rat vs. guinea pigs).

The present study observed a negative optical signal in V1, as well as a positive signal followed by a negative signal in LV and RV, to auditory stimulation. Compared to the DCB response, responses in the visual areas were weak. One may suspect that the negative responses in the visual areas are intrinsic optical signals. This is unlikely because we used only a single stimulus to evoke the response, and the responses returned to baseline level in less than 200 ms. Voltage-sensitive dye signals have been previously shown to be linearly related to membrane potential [41]. Therefore, the small response amplitudes in the visual areas could be attributable to sub-threshold auditory input in the visual cortex. Alternatively, because the optical signal in each pixel reflected activity in all cells or cellular processes covered by the pixel, the small signal amplitude could also be explained by supra-threshold activity in a small number of neurons. Previous anatomical evidence of a sparse projection from the auditory cortex to the V1 [18], [19], [20], [21] is consistent with the present observations. Our results, however, should not be taken as evidence that these projections were functional. Further studies that include manipulation of the projections are needed to demonstrate if they are functional.

Because the voltage-sensitive dye RH-795 converts membrane depolarization to a reduction in fluorescence, which is depicted here as a positive signal, a positive response might reflect excitation and a negative change might represent inhibition. A typical voltage-sensitive dye signal from a cortical sensory response is a positive change followed by a negative phase [22], [25], [26], [34], [42], as demonstrated here for the visual response in V1 and the auditory response in the AC. Horikawa et al. showed that acoustically evoked positive changes in the auditory cortex are blocked by glutamatergic antagonists, and the negative signal following the positive signal is eliminated by GABAergic antagonists [33]. In the present study, acoustically evoked optical signals in V1 exhibited only a negative change without a preceding positive phase (see Fig. 4). This result may suggest that auditory input to V1 was inhibitory. Although long-range cortico-cortical GABAergic projections have been shown in several recent studies (see Ref. [43] for review), it is unclear whether projections from the auditory cortex to V1 [18], [19], [20], [21] are GABAergic or not. Alternatively, the negative V1 response could be attributed to action of cortical intrinsic inhibitory interneurons, which ramify axons within the cortex to inhibit other neurons [44]. If this were the case, positive phase reflecting interneuron excitation should precede the negative response signal, although the positive phase might be too weak to detect. These issues await clarification in future studies that directly measure membrane potentials at the cellular level.

In light of previous findings that activity evoked in the somatosensory cortex propagates to the motor cortex [45], and that bidirectional activity propagation occurs between visual and somatosensory cortices via parietal association area [35], it is also possible that auditory responses observed here in the visual areas might be mediated by cortical regions other than sensory areas.

### Visuo-auditory Interaction in Visual Areas

In all visual and auditory fields identified in the present study, significant visuo-auditory interactions were observed in the simultaneous condition. Interactions appeared as enhanced V1 visual responses as a result of auditory stimulation, which is consistent with previous findings in humans [12]. The observation that visuo-auditory interactions occurred in V1 earlier than in other fields, (see Fig. 5), suggested that visuo-auditory interactions in V1 were not due to feedback from higher order areas, but instead originated within V1. It is possible that because not all extrastriate visual areas were analyzed, the possibility of feedback from higher order areas was not completely excluded. Wallace et al. showed that the strongest bimodal visuo-auditory response and the strongest visuo-auditory interaction were observed in areas between V1 and the primary auditory area [46]. The LV area identified in the present study is such an area. The later appearance of a visuo-auditory interaction in LV, compared to V1, strongly suggests that V1 was the cortical area where the visuo-auditory interaction originally occurred. In the acoustic leading condition, significant visuo-auditory interactions were detected only in V1, which argues in favor of this hypothesis. The dependence of multisensory interactions on the relative onset timing of cross-modal stimuli has been reported previously [46].

In their seminal work, Giard and Peronnet [2] studied visuo-auditory interactions in humans through the use of electroencephalography (EEG) recordings, demonstrating that the {AV}− ({V}+{A}) signal can exhibit a latency as short as 40 ms at occipital recording sites. This short latency led the authors to conclude that visuo-auditory interactions occur at the early stage of cortical visual processing [2]. Similar EEG observations have been performed in a number of other studies [47], [48], [49]. However, in the present study, the onset time of visuo-auditory interactions was 60 ms (see Fig. 5), which appeared to be too long, especially considering the small size of the guinea pig brain and the report that visuo-auditory interactions in human V1 occur with an onset time of 40 ms [2], [47], [48], [49]. Recently, Raij et al. [12] demonstrated in a human MEG study that the onset time of visuo-auditory interaction is 74 ms in V1 and 80 or 85 ms in the primary auditory cortex. The authors also used fMRI to support the MEG source localizations. The 60 ms onset time of visuo-auditory interactions in guinea pig V1 found here, is thus consistent with human studies [12]. The short onset time reported in previous EEG studies [2], [47], [48], [49] is likely attributable to the contribution of EEG signals from subcortical visuo-auditory interactions [12]. Unlike EEG and MEG signals, both of which exhibit inherent ambiguity in source localization, the voltage-sensitive dye optical signals in the present study reflected local activity of cortical neurons covered by each pixel. There is strong evidence that voltage-sensitive dye staining is limited to the cortex [25], [50].

The earlier onset of visuo-auditory interaction in the V1 compared to other areas in the simultaneous condition (see Fig. 5) may suggest that the V1 interaction propagated to other areas. The absence of interactions in the acoustic leading condition in areas other than the V1, however, suggests that the propagation of interaction, even if existed, was not an autonomous process, but rather depended on the relative onset timing of cross-modal stimuli. It is also possible that visuo-auditory interaction occurred independently in each cortical area. Selective manipulation of activities in specific cortical areas is needed in future studies to better understand these mechanisms.

The auditory responses in LV and DCB had earlier onset times than in V1 (see Fig. 5C, D). This raises the possibility that auditory responses in V1 might have been affected by auditory responses in LV and DCB, as well as in A1. One may suspect that a visuo-auditory interaction in V1 is a result of interaction in these fields. Our data argue against this possibility, because the interaction occurred earlier in V1 than in other fields. Visuo-auditory interaction did not occur at the response onset, but at later times close to the positive peak of visual responses or the negative peak of auditory responses, (see Fig. 5C). The late occurrence of visuo-auditory interaction does not allow conclusive argument about the underlying pathways based on response onset latencies to unimodal stimulation.

Although the visuo-auditory interactions observed in the present study were significant in all cortical areas examined, the magnitude of the interactions was moderate in the visual areas. Nevertheless, these results are consistent with previous studies. In humans, for example, the V1 signal ({AV}−({A}+{V}) also appears to be small [12], although it has not been quantified. In monkey V1, the {AV} response exhibits shorter latencies than {V}, but the two responses are not statistically different [10]. The absence of change in response magnitude in monkeys is likely due to the fact that Wang et al. [10] used the unit recording technique that does not detect sub-threshold responses. The MEG signals recorded in humans [12], as well as the optical signals recorded in the present study, included sub-threshold activities. Wang et al. [10] also made an interesting observation that visuo-auditory interaction occurs only when the animal gazes at the stimulus. The present experiments were performed in anaesthetized animals. It is possible that visuo-auditory interactions in guinea pigs could be enhanced if the animal was awake and attentive to the stimulus.

In summary, by exploiting high spatial and temporal resolution of voltage-sensitive dye imaging, the present study has demonstrated that visuo-auditory interaction in V1 occurs earlier than other visual areas in guinea pigs. These results suggest a V1-origin for cortical visuo-auditory interaction. Future studies should include recordings of auditory responses at the membrane potential level to better understand the cellular mechanisms of visuo-auditory interactions in V1. Because visuo-auditory interactions occurred in multiple cortical fields of visual and auditory modality, the relationship between activity in each of these areas and visuo-auditory interaction at the recognition level is an essential question to address in future studies.

## Materials and Methods

### Ethics Statement

All experiments were performed according to the Guidelines for Use of Animals in Experiments of Kumamoto University. The protocol was approved by the Committee of Animal Experiments of Kumamoto University.

### Animal Preparation for Optical Imaging

Experiments were performed as previously described by our group [25], [26]. Adult guinea pigs weighing 300–700 g were used in the present study. The majority of guinea pigs were albino, but three pigmented animals were also utilized to test the V1 auditory response. Anesthesia was induced with a mixture of ketamine (46 mg/kg) and xylazine (24 mg/kg), and anesthesia was maintained by injecting half of the initial dose per hour during surgery. During recording, a quarter of the initial dose was injected per half hour to reduce fluctuations in anesthetic levels. Prior to surgery, atropine sulfate (0.2 mg/kg) was administered to suppress bradyarrhythmia and bronchial secretions, and dexamethasone (0.5 mg/kg) was administered to suppress brain edema. The guinea pigs were cannulated for artificial respiration during recording. CO_2_ levels of expiratory flow and heart rate were monitored throughout the experiment. Rectal temperature was maintained at 38.0±0.5°C. The skull over the left visual cortex was removed with reference to the sagittal suture and the lambdoid suture. The medial edge of the exposed cortex was approximately 10 mm in length and was made parallel to the midline with a distance of 1.5 mm; the caudal edge was about 9 mm. The mediocaudal corner of the exposed cortex reached the lambdoid suture. Following resection of the dura mater, the cortex was stained twice with the voltage-sensitive dye RH-795 (0.7 mg/ml in saline; Invitrogen, Grand Island, NY, USA) for 45 minutes each time. The ipsilateral eye was closed and covered with a small metal dome to prevent stimulation to the eye by the excitation light used for imaging. Optical signals were sampled at 500 Hz or 1 kHz using a CMOS imaging system (MiCAM Ultima, Brainvision, Tokyo, Japan). The recording field was 6.25×6.25 mm^2^, and one edge of the recording field was made parallel to the midline; the medial edge was about 2.5 mm to the midline and the caudal edge was set slightly rostral (∼0.5 mm) to the cross point between the sagittal suture and the lambdoid suture.

### Visual and Acoustic Stimuli

Light-emitting diodes (LEDs) were used to present light flash stimuli. Each flash of light lasted 10 ms. To minimize interference with the imaging system, the LED was attached to one end of an opaque cylinder, and the other cylinder end was attached to the skin around the contralateral eye to confine light stimulation to the eye. LEDs were powered by custom-made constant current circuits [42], and controlled and triggered by TDT RX5 and RA16. To test visuo-auditory interactions, a low-light intensity of 50 Lux was used to increase the possibility of observing visuo-auditory interactions [51]. All acoustic stimuli were digitally generated at a rate of 195 kHz using custom-made software, and loaded onto a TDT real-time processor (RX6, Tucker-Davis Technologies, Alachua, FL, USA). The sound pressure level (SPL) was calibrated using a sound level meter (type 2610 with a model 4191 microphone, Brüel & Kjær, Nærum, Denmark). Broadband noises (bandwidth: 125–32,000 Hz, 50-ms duration, 10-ms on/off cosine ramp, 30-ms plateau) were presented at 70–75 dB SPL to both ears *via* earphones (ATH-C602, Audio-Technica, Tokyo, Japan) driven by a headphone buffer (HB7, Tucker-Davis Technologies, Alachua, FL, USA). The noise presented to one ear was made independent to that presented to the other ear, to remove spatial information from auditory stimuli. During recording of auditory responses in the cortex, the contralateral eye was light-adapted at 300 Lux using an attached LED. Light adaptation was not performed in the visuo-auditory interaction analysis.

For analysis of visuo-auditory interaction in the visual cortex, sets of responses to a visual stimulus {V}, responses to a acoustic stimulus {A}, and responses to concurrent acoustic and visual stimuli {VA} were recorded. The presentation order of the visual stimulus, acoustic stimulus, and concurrent acoustic and visual stimuli was systematically interleaved to minimize the effect of response fluctuation during the experiment.

Heartbeat and respiration has been shown to significantly interfere with optical *in vivo* recording [52]. Therefore, to prevent this interference, artificial ventilation was temporarily stopped (<0.8 sec for each trial) and recording was synchronized with the heartbeat by recording electrocardiogram (ECG) and using the ECG signal to control the timing of optical recording. Evoked responses were obtained by subtracting recordings without stimulus presentation from recordings with stimulus presentation [25], [32]. The inter-recording interval was more than 4 seconds.

### Data Analysis for Optical Imaging

Optical signals were initially low-pass filtered using a two-dimensional Gaussian-windowed sinc filter (<0.4 cycle/pixel; spatial resolution; ∼160 µm in diameter). Fractional changes in fluorescence (ΔF/F_0_s) in each pixel were then calculated from the spatially filtered signals using an average of 50 frames prior to stimulus onset (F_0_), followed by filtering with one-dimensional band-pass (4–125 Hz) Gaussian-windowed sinc filter. The amplitude of ΔF/F_0_ was color-coded to show the spatial response patterns.

To represent responses from each animal, an ROI was set for each cortical area. The ROI shape was a circle, and the ROI center was set to the epicenter of responses in each area (Fig. 2A). For ROI analysis, the spatial filter was not applied to focus on responses in the area. Optical signals in the ROI were averaged in each animal.
